# Development of a Multivariable Risk Prediction Tool to Predict Adverse Outcomes among Children with Type 1 Diabetes: A Pilot Study

**DOI:** 10.1155/2024/8335604

**Published:** 2024-05-20

**Authors:** Fiona Lieu, Wrivu N. Martin, Stewart Birt, Joerg Mattes, Richard G. McGee

**Affiliations:** ^1^The Central Coast Clinical School, School of Medicine and Public Health, The University of Newcastle, Callaghan, New South Wales, Australia; ^2^School of Medicine and Public Health, The University of Newcastle, Callaghan, New South Wales, Australia; ^3^Department of Paediatrics, Gosford Hospital, Central Coast Local Health District, Gosford, New South Wales, Australia; ^4^Priority Research Centre GrowUpWell, Hunter Medical Research Institute, Newcastle, New South Wales, Australia; ^5^Department of Paediatrics, Campbelltown Hospital, South West Sydney Local Health District, Campbelltown, New South Wales, Australia

## Abstract

**Background:**

Children and adolescents with type 1 diabetes mellitus (T1DM) are frequently hospitalised for severe hypoglycaemia, hyperglycaemia, and diabetic ketoacidosis (DKA). While several risk factors have been recognised, clinically identifying these children at high risk of acute decompensation remains challenging.

**Objective:**

To develop a risk prediction model to accurately estimate the risk of acute healthcare utilisation due to severe hypoglycaemia, hyperglycaemia, and DKA in children and adolescents with T1DM.

**Materials and Methods:**

Using a retrospective dataset, baseline demographic and clinical data were collected from patients (<18 years) seen at a regional paediatric diabetes clinic from 1 January 2018 to 1 January 2020. The outcome was the number of emergency department presentations or hospital admissions for severe hypoglycaemia, hyperglycaemia, and DKA across the study period. Variables that were significant in univariate analysis were entered into a multivariable model. Receiver operator characteristic (ROC) curves assessed the model's discrimination and generated cut-offs for risk group stratification (low, medium, and high). Kaplan–Meier survival analysis measured time to acute healthcare utilisation across the risk groups.

**Results:**

Our multivariable risk prediction model consisted of five predictors (continuous glucose monitoring device, previous acute healthcare utilisation, missed appointments, and child welfare services involvement and socioeconomic status). The model exhibited good discrimination (area under the ROC = 0.81), accurately stratified children into low-, medium-, and high-risk groups, and demonstrated significant differences between median time to healthcare utilisation.

**Conclusion:**

Our model identified patients at an increased risk of acute healthcare utilisation due to severe hypoglycaemia, hyperglycaemia, and DKA.

## 1. Introduction

Over 1.1 million children and adolescents live with type 1 diabetes mellitus (T1DM) worldwide [1, 2]. Severe hypoglycaemia and diabetic ketoacidosis (DKA) are preventable, life-threatening complications of this disease. DKA is the leading cause of death in children and adolescents with T1DM globally, with an annual mortality rate of 0.15%–0.51% [3–7]. In Australia, 22.4% of children with T1DM will be hospitalised due to DKA, and 10% of these children will be hospitalised more than once for this complication in their lifetime [8]. Severe hypoglycaemia can precipitate seizures and comas [9–11]. On average, patients with T1DM experience this complication once to twice per year [12], with the highest risk among young children [13, 14].

Hospitalisation due to these acute issues causes a range of biopsychosocial effects. Firstly, being hospitalised for disease complications is emotionally distressing to children and adolescents [3, 12, 15]. Repeated hospital admissions disrupt school attendance and impair the child's and their family's quality of life [12, 16, 17]. Hospital attendance and admission are also costly to the community due to interruptions to carer employment [8], long average lengths of stay [12], and frequent use of high dependency or intensive care units [3, 8, 12]. Preventing these complications with timely intervention is crucial to reducing the burden of this disease on children, their parents, and the community at large.

Unfortunately, identifying children at high risk of these complications remains challenging. While several biological [18–22], anthropomorphic [21–30], and sociodemographic [8, 25, 27, 31–33] risk factors are associated with these complications, there is currently no reliable way of using these data to estimate a child's personalised risk. Previous attempts to do this have had important limitations. For instance, Schwartz et al. [34] developed a psychosocial risk index for poor glycaemic control, diabetes-related ED presentations, and DKA episodes. However, this model was limited to sociodemographic and psychosocial variables. Other factors influencing T1DM outcomes were not examined, such as the type of insulin therapy or patterns of glycaemic monitoring. Another study by Glick et al. [35] developed a risk screening tool to determine a child's baseline risk of experiencing DKA or hypoglycaemia at the time of their T1DM diagnosis. Again, this study only assessed sociodemographic or psychosocial risk factors; moreover, its efficacy in predicting risk at any point *after* diagnosis remains unclear. Finally, Mejia-Otero et al. [33] developed a model for predicting episodes of DKA among children with T1DM; however, it was limited to only predicting DKA episodes and similarly did not investigate the predictive value of specific indicators of glycaemic control.

As the prevalence of childhood T1DM and its attendant complication rises [1, 36], an accurate tool derived from sociodemographic *and* clinical variables is needed to predict and prevent acute healthcare utilisation in children with T1DM. Therefore, the aim of this study was to develop a clinical risk prediction tool to accurately estimate the risk of acute healthcare utilisation due to severe hypoglycaemia or DKA in children and adolescents with established T1DM.

## 2. Methods

We report our findings according to the Transparent Reporting of a Multivariable Prediction Model for Individual Prognosis or Diagnosis (TRIPOD) guidelines [37].The Hunter New England Human Research Ethics Committee granted this project ethics approval (2021/PID00346).

### 2.1. Data Sources

We collected data by conducting a retrospective cohort study of the paediatric diabetes clinic in the Central Coast Local Health District (CCLHD). The CCLHD is a large, geographically distinct region in Australia. The paediatric diabetes clinic is the *only* public paediatric diabetes service in the region and provides care to all children with diabetes in the district. The clinic provides care to approximately 160 children and adolescents with T1DM and is staffed by two paediatricians, diabetes nurse educators, dietitians, and one social worker.

Children were included in this study if they (1) were aged between 1 and 17 years, (2) had a duration of T1DM of more than 9 months at the time of enrolment, and (3) were seen at the CCLHD paediatric diabetes clinic between 1 January 2018 and 1 January 2020. We excluded patients from our study if they (1) had a new diagnosis of T1DM with positive IAA, IA2, or GAD antibody levels (less than 9 months at the time of enrolment), (2) had another form of diabetes (as suggested by antibody levels, genetic testing, or clinical history) or (3) were transitioning to the young adult diabetes clinic. Nine months was chosen as the average period of the “honeymoon” phase [38].

We extracted baseline and ongoing clinical data from the electronic medical records (EMR) of participants. Baseline was defined as their first routine clinic visit after 1 January 2018. We then extracted the data from each clinic visit until 1 January 2020 and observed participants from study enrolment to 31 December 2020 to identify hospital presentation or admission for diabetes-related complications.

### 2.2. Outcome

The primary outcome of our model was acute healthcare utilisation, defined as emergency department (ED) presentations or hospital admissions for (1) DKA, (2) hyperglycaemia, or (3) severe hypoglycaemia, defined as a low blood glucose event with severe cognitive impairment needing external assistance to perform corrective actions in keeping with the 2018 ISPAD clinical practice consensus guidelines [39]. Participants who had planned hospital admissions for glycaemic stabilisation were excluded (*n* = 5). We manually reviewed all ED presentations and hospital admissions to confirm that the presentation was due to DKA, hyperglycaemia, or severe hypoglycaemia as defined by the 2018 ISPAD Clinical Practice Consensus Guidelines [39].

### 2.3. Predictors

Predictors for acute diabetic outcomes were identified based on input from the CCLHD paediatric diabetes team and a systematic review of the literature (Supplementary file 1). Supplementary file 1 lists the definitions, data sources, and timing of collection for each predictor variable. Predictors were classified into modifiable and non-modifiable categories.

Non-modifiable predictors included gender, current age, age at diagnosis, duration of diabetes, socioeconomic status, acute healthcare utilisation in the previous 12 months, presence of neuropsychiatric comorbidities, and any involvement with the Department of Communities and Justice (DCJ), which is a state department that provides child protection services. Socioeconomic status (SES) was coded based on the Index of Relative Socioeconomic Advantage and Disadvantage (IRSAD) quintiles, which summarise the economic and social conditions of residents within an area based on the 2016 Australian Bureau Statistic calculator [40]. An IRSAD score of one indicates “most disadvantaged”, and five indicates “least disadvantaged”. Acute healthcare utilisation in the previous 12 months due to DKA, hyperglycaemia with ketosis, or severe hypoglycaemia was coded as “none”, “one or two”, and “more than two”. The presence of neuropsychiatric comorbidities (defined as neurodevelopmental and psychiatric disorders) was coded as “none”, “one or two”, or “more than two” based on the manual review of EMR. Involvement with DCJ was extracted from the diabetes social worker notes and coded as “no involvement, ever”, “previous involvement”, and “current involvement”.

Modifiable predictors included glycosylated haemoglobin (HbA1c), number of missed clinic visits in the previous 6 months (any scheduled appointment not attended, with on average an appointment scheduled once every 3 months), method of insulin administration, whether the child wore a continuous glucose monitoring device (CGM) with hyperglycaemia or hypoglycaemia alarms, and average number of blood glucose monitoring checks per day. Since April 2017, the Australian Government has fully funded CGM products for young people living with T1DM aged <21 years through a national scheme. Glycaemic monitoring parameters varied depending on the mode of monitoring. For participants who wore CGM, we extracted their percentage of time in range, percentage of wear time, and the number of calibrations per day. For participants who wore insulin pumps, we extracted the number of insulin boluses delivered per day and the average number of days between site changes. Insulin pumps are currently not funded by the Australian Government, and patients usually obtain them via private health insurance cover or the JDRF Insulin Pump Program for children who meet the financial and clinical eligibility requirements. All the insulin pumps used by participants in this study had a low-glucose suspension option. HbA1c was collected as part of routine clinic management using a DCA Vantage HbA1c analyser. HbA1c values were recorded for each clinic visit during their follow-up period and an average generated; values greater than 14 (i.e., above the range of the DCA Vantage assay) were recorded as 14. The number of missed clinic visits in the previous 6 months was extracted from EMR and classified as “none”, “one”, or “two or more”. CGM was coded as “yes” if the child wore CGM frequently (≥50% wear time) or “no” if the child did not have CGM or was recorded in the participant's EMR as infrequently wearing CGM (<50% wear time). The average number of blood glucose monitoring checks was only recorded for children who did not wear CGM and instead performed fingerstick blood glucose monitoring. This value was extracted from their blood glucose meter download during their clinic appointment and recorded in the participant's EMR. The method of insulin administration was defined as “multiple daily injections” or “insulin pump”. CGM and insulin pump parameters were extracted from each device's download, uploaded to the child's EMR at every clinic appointment.

### 2.4. Missing Data

We planned to handle missing data with complete case analysis.

### 2.5. Statistical Analysis

We summarised data as absolute numbers and percentages for categorical variables, means with standard deviations for normally distributed continuous variables, and medians with interquartile ranges for non-normally distributed variables.

To estimate the relationship between our predictors and outcomes, we conducted univariate analyses using logistic regression to estimate the effect of a single unit change in our predictor on the odds of experiencing an acute diabetic complication during our study period. We accepted a *p* value of <0.05 as significant and did not adjust for multiple testing, as the primary purpose of our study was hypothesis generation. Variables that were significant predictors in the initial analyses were then included in a multivariable logistic regression where the outcome was any acute healthcare utilisation due to acute diabetic complications. We then sequentially removed variables that contributed <1% to the model's pseudo-*R*-squared value to generate the most parsimonious multivariable model.

We applied our final multivariable equation to each child in our cohort. This generated a baseline score for each child. We then generated receiver operator characteristic (ROC) curves to assess the sensitivity and specificity of the model at predicting acute healthcare utilisation due to acute diabetic complications during the entire study duration, as well as at 6, 12, 18, and 24 months from baseline. Using the cut-offs derived from our full study duration ROC curve, we further divided our cohort into three groups: (1) low risk, who had scores below the 95% sensitivity threshold (*n* = 25); (2) high risk, who had scores above the 90% specificity threshold (*n* = 27); and (3) moderate risk, who had scores between these thresholds (*n* = 51).

We also generated Kaplan–Meier survival curves and conducted Cox regression to compare time to acute healthcare utilisation among risk groups. We assessed proportional hazard assumption both graphically and numerically and performed log-rank tests to assess the equality of survivor functions across our risk groups. We then generated both mean and median time to acute healthcare utilisation for each of these groups.

Additionally, we conducted repeated measures analyses using linear mixed models and an unstructured covariance matrix. We set HbA1c as our outcome and categorical risk factors as our exposures to assess these relationships *within* participants over time. Analyses were adjusted for age, sex, and study duration.

Finally, we conducted negative binomial regression to assess the relationship between risk groups and the number of acute healthcare utilisation episodes. Although we observed several zero-counts, we found no evidence that this was secondary to inflation. We used negative binomial regression instead of a standard Poisson regression because the variance of our outcome was higher than the mean. We conducted all analyses on STATA 15.

## 3. Results

There were 103 patients aged 1–17 years with established T1DM observed at the CCLHD paediatric diabetes clinic between 1 January 2018 and 1 January 2020 who met the inclusion criteria. Thirty-nine patients presented with acute healthcare utilisation during this time. The baseline demographic and clinical characteristics of these patients are summarised in Table 1. In general, the cohorts that experienced acute healthcare utilisation had a slightly higher baseline HbA1c, were more likely to use multiple daily injections, were less likely to use CGM, had more acute healthcare utilisation episodes in the 12 months preceding baseline, had more missed clinic appointments, had more neuropsychiatric comorbidities, were more likely to have an active DCJ case, and were more likely to be in the lowest IRSAD quintile. Both cohorts were similar in age, gender, age at onset, and duration of diabetes. Overall, there was complete information about the predictors and outcomes for our analysis.

### 3.1. Model Development

Eight individual predictors (*baseline HbA1c*, *insulin administration method*, *CGM device worn*, *previous acute healthcare utilisation*, *missed clinic visits*, *DCJ involvement*, *SES*, and *number of insulin boluses per day*) were significant in univariate analyses for DKA, severe hypoglycaemia, or acute healthcare utilisation (Table 2). For DKA, significant predictors included elevated baseline HbA1c (*β* = 0.57; 95% CI, 0.24–0.90; *p*  < 0.01), no CGM wear (CGM wear, *β* = −1.17; 95% CI, −2.27 to −0.07; *p*=0.04), two or more episodes of acute healthcare utilisation in the last 12 months (*β* = 3.19; 95% CI, 1.71–4.67; *p* < 0.01), and an active DCJ case (*β* = 2.64; 95% CI, 1.16–4.11; *p* < 0.01). Only participants on multiple daily insulin injections (instead of insulin pump) were significantly associated with severe hypoglycaemia (insulin pump, *β* = −2.29; 95% CI, −4.41 to −0.17; *p*=0.03). Parameters associated with acute healthcare utilisation were elevated baseline HbA1c (*β* = 0.40; 95% CI, 0.11–0.69; *p*  < 0.01), multiple daily insulin injections (insulin pump, *β* = −0.85; 95% CI, −1.66 to −0.03; *p*=0.04), no CGM device (CGM wear, *β* = −1.06; 95% CI, −1.90 to −0.22; *p*=0.01), multiple hospitalisations in the last 12 months (*β* = 2.49; 95% CI, 0.90–4.08; *p*  < 0.01), increased number of missed clinic visits in the last 6 months (*β* = 1.01; 95% CI, 0.27–1.76; *p*  < 0.01), active DCJ case (*β* = 2.05; 95% CI, 0.44–3.66; *p*=0.01), low SES (2nd IRSAD quintile, *β* = −1.27; 95% CI, −2.49 to −0.06; *p*=0.04), and reduced number of insulin boluses per day (*β* = −0.40; 95% CI, −0.77 to −0.04; *p*=0.04).

In addition to study duration, these eight variables were entered into multivariable analysis. The final parsimonious model retained five predictors: CGM wear (*β* = −0.86; 95% CI, −1.92–0.21; *p*=0.12), acute healthcare utilisation in the previous 12 months (1 event, *β* = 1.80; 95% CI, 0.07–3.53; *p*=0.04; ≥2 events, *β* = 2.87; 95% CI, 0.52–5.22; *p*=0.02), number of missed clinic visits in the previous 6 months (1 visit, *β* = 0.70; 95% CI, −1.02–2.43; *p*=0.43; ≥2 visits, *β* = 2.38; 95% CI, 0.06–4.70; *p*=0.04), active involvement with DCJ (*β* = 1.61; 95% CI, −0.26–3.49; *p*=0.09), and SES (see Table 3). Previous acute healthcare utilisation and number of missed clinic visits remained significant in multivariable analysis. The final model accounted for 26% of the variance in children who experience diabetes-related acute healthcare utilisation.

### 3.2. Model Assessment: Receiver Operator Characteristic Curves

Table 4 indicates the area under the curve (AUC) values for the model's discrimination at predicting acute healthcare utilisation at 6, 12, 18, and 24 months and the full study period (906 days). The model indicated low discrimination at 12 months (AUC = 0.67; 95% CI, 0.55–0.78) and moderate discrimination at 6 (AUC = 0.79; 95% CI, 0.64–0.94), 18 (AUC = 0.79; 95% CI, 0.69–0.89), and 24 months (AUC = 0.78; 95% CI, 0.68–0.89) and performed its best for the full study period (AUC = 0.81; 95% CI, 0.72–0.90).

**Figure 1:**
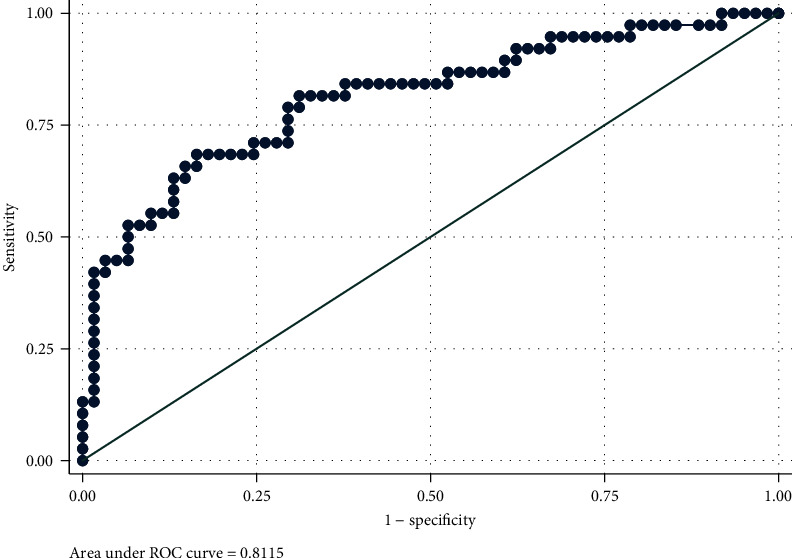
ROC curve for diagnostic ability to predict acute healthcare utilisation.

The ROC curve of the final model (Figure 1) provided values that corresponded to a 95% sensitivity and 90% specificity for predicting acute healthcare utilisation during the full study period. These equated to scores of 0.17 and 0.49, respectively. We therefore defined the model's risk groups as follows: (1) low-risk group for acute healthcare utilisation, participants with a score of less than 0.17; (2) high-risk group for acute healthcare utilisation, participants with a score greater than 0.49; and (3) moderate-risk group for acute healthcare utilisation, participants with scores between 0.17 and 0.49.

### 3.3. Survival Time Analyses

**Figure 2:**
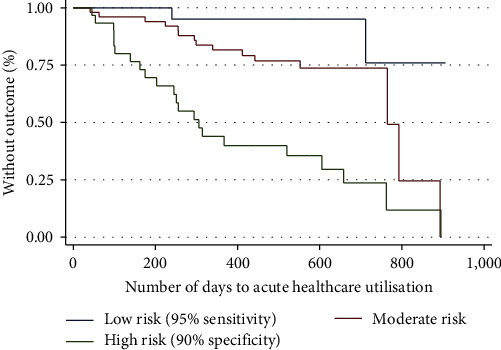
Kaplan–Meier survival curves for time to acute healthcare utilisation by risk groups.

Cox regression of our survival data showed that participants in the high-risk group were 11.7 (95% CI, HR 2.75–49.99) times more likely to experience acute healthcare utilisation compared to low-risk participants (Table 5). Similarly, moderate-risk participants experienced acute healthcare utilisation 3.5 times more frequently (95% CI, HR 0.80–15.38), though this result was not statistically significant. We assessed the proportional hazard assumption numerically (proportional hazards test, *p*=0.40) and visually, which suggested that the assumption was met. The differences in time to acute healthcare utilisation between risk groups are clearly illustrated in Figure 2. Low- and moderate-risk groups experienced a significantly longer time to acute healthcare utilisation compared to high-risk participants. The median time to acute healthcare utilisation was 295 days for participants in the high-risk group (95% CI, 175–605) and 765 days in the moderate-risk group (95% CI, inestimable) and could not be ascertained for the low-risk group, as a significant proportion of these participants did not experience the primary outcome during the study duration.

### 3.4. Negative Binomial Regression: Number of Acute Healthcare Utilisation Episodes

We also conducted negative binomial regression to determine the relationship between our risk groups and the number of acute healthcare utilisation episodes. Our results showed that even when age, study duration, sex, and baseline HbA1c were controlled, the incidence rate ratios for acute healthcare utilisation were 6.94 (95% CI, 1.37–35.26) to 35.5 (95% CI, 6.48–194.55) times higher in moderate- and high-risk groups, respectively, compared to the low-risk group (Table 6).

### 3.5. Repeat Measures Analyses: Linear Mixed Models

**Figure 3:**
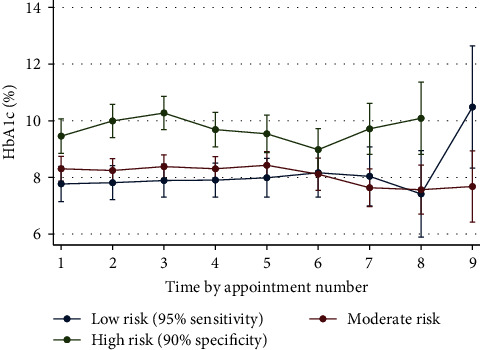
HbA1c trends by multivariable risk groups.

**Figure 4:**
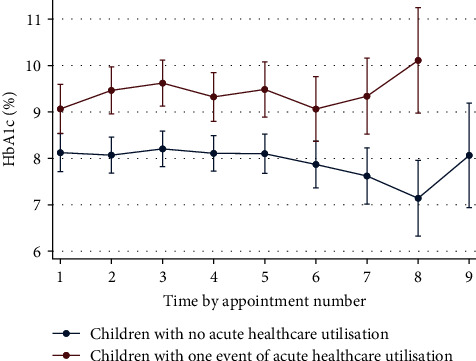
HbA1c trends by acute healthcare utilisation.

We generated linear mixed models adjusted for age, gender, and study duration to examine the trajectories of glycaemic control within our risk groups over time (Supplementary file 1).  Figure 3 demonstrates that compared to low-risk participants, high-risk participants had consistently higher mean HbA1c readings over time (*β* = 1.54; 95% CI, 0.72–2.36). These differences were more pronounced between the 1st to 3rd and 6th to 8th appointments, though only the former was statistically significant. We then examined each of the variables that comprise the score with linear mixed models. These models showed that the mean HbA1c was persistently lower among participants wearing CGM at baseline (*β* = −1.06; 95% CI, −1.72 to −0.43; Supplementary file 1). Contrastingly, the mean HbA1c was persistently higher among participants with acute healthcare utilisation in the 12 months preceding baseline (*β* = 1.66; 95% CI, 0.62–2.68; Supplementary file 1) as well as participants with an active DCJ case at baseline (*β* = 1.70; 95% CI, 0.60–2.82; Supplementary file 1). Over time, the differences between groups diminished. We found no consistent relationships between SES and HbA1c (data not presented). Finally, we compared HbA1c over time between participants with and without acute healthcare utilisation during our study period; as expected, participants with acute healthcare utilisation had persistently higher HbA1c, an effect that was amplified with time (*β* = 0.94; 95% CI, 0.28–1.60; Figure 4).

## 4. Discussion

Children and adolescents with T1DM are at an increased risk of being hospitalised for acute, life-threatening diabetic complications. Early identification of children at greater risk of experiencing these preventable complications may allow for early targeted intervention. This could reduce the morbidity, cost, and risk of mortality associated with diabetic decompensation. While several risk factors are associated with developing these complications, there is currently no unified tool that synthesises these risk factors into an individualised risk score for children with T1DM. The main aim of our study was, therefore, to develop a tool to predict a child's risk of requiring ED presentation or hospital admission for DKA, hyperglycaemia, or severe hypoglycaemia using a simple suite of known risk factors.

### 4.1. Comparison with Existing Literature and Novel Risk Factor Findings

Our univariate analyses of risk factors for acute diabetic complications were largely consistent with existing literature (Supplementary file 1). In our analyses, lower baseline HbA1c [18, 22, 27, 29, 33, 41, 42], use of an insulin pump [25], no acute healthcare utilisation in the previous 12 months [22, 33], a lower number of missed clinic visits [21], and higher SES [31] were each associated with a reduced risk of acute diabetic complications (see Table 2). We observed inconsistent relationships between gender, age, and acute diabetic complications; this result conforms with the existing literature, which itself reports mixed findings (Supplementary file 1).

We also contribute several novel observations. Firstly, we observed that CGM was associated with a reduced risk of acute healthcare utilisation; in our study, children who wore CGM were 1.17 times less likely to experience DKA (95% CI, −2.27 to −0.07; *p*=0.04) and 1.06 times less likely to require acute healthcare utilisation (95% CI, −1.90 to −0.22; *p*=0.01). These children also had lower mean HbA1c values over time (Supplementary file 1). While CGM wear has previously been correlated with optimised glycaemic variability in children and adolescents [43, 44] and reduced hospitalisation rates in *adults* with T1DM [45], our observed association between CGM wear and reduced acute diabetes-related healthcare utilisation is novel among children and adolescents with T1DM. An important explanation for this finding could be that CGM technology provides real-time feedback about glycaemic control [46–48]. Instant access to blood glucose levels may, therefore, enhance the ability of wearers or carers to make effective decisions around insulin dosing or diet [49]. We also observed that an increased number of missed appointments was associated with an increased risk of acute healthcare utilisation; this association remained significant across univariate and multivariate analyses. Reasons for missing appointments may be complex and multifactorial, incorporating several unobserved biopsychosocial factors [50–53].

We also contribute novel observations regarding sociodemographic predictors of diabetic decompensation. In our study, children with active DCJ involvement were more than twice as likely to experience DKA (*β* = 2.64; 95% CI, 1.16–4.11; *p* < 0.01) and present to ED or be hospitalised for acute diabetic complications (*β* = 2.05; 95% CI, 0.44–3.66; *p*=0.01). To our knowledge, this has not been explored elsewhere in the literature. While we could not identify any specific mechanistic link to glycaemic control, this reflects another sociodemographic variable that may have long-term consequences on healthcare outcomes among children with T1DM. Australian socioeconomic status, defined by the Index of Relative Socioeconomic Disadvantage (IRSAD), was another novel predictor of acute healthcare utilisation. In our study, we observed that participants from higher IRSAD quintiles were less likely to experience acute healthcare utilisation compared to participants in the most disadvantaged (1st) IRSAD quintile. The difference was most pronounced among the 2nd quintile for acute healthcare utilisation (*β* = −1.27; 95% CI, −2.49 to −0.06; *p*=0.04). Contrastingly, children from the most advantaged IRSAD quintile exhibited a similar risk of diabetic ketoacidosis to those from the lowest quintile (*β* = 0.10; 95% CI, −1.65–1.84); for this outcome, IRSAD followed a “U-shaped” pattern. While socioeconomic disadvantage is a known risk factor for acute diabetic complications [21, 31], our findings will require replication in larger cohorts. Nonetheless, we contribute important data examining the association between an Australian SES index and a child's risk of adverse diabetic outcomes.

In addition to these findings, we also made observations contrary to our initial hypothesis. First, several well-established variables were uninformative or insignificant in our final multivariable model; these included baseline HbA1c, wearing an insulin pump, and the number of daily boluses delivered via an insulin pump. Although these variables were significant independent predictors of acute diabetic complications in univariate analyses, our sample size may have been too small to detect significant differences in a multivariable model with several other variables. Alternatively, simultaneous adjustment for variables linked to these risk factors may have diminished their individual effect; this would explain why, respectively, each of these variables contributed less than 1% to our final model's *R*-squared value in the presence of other explanatory variables. Another unexpected finding was the lack of association between our predictors and severe hypoglycaemia; only insulin pump therapy was significant in univariate analyses. Again, this may be due to the small number of participants who experienced this outcome in our cohort (*n* = 9). Nonetheless, a child's risk for severe hypoglycaemia was significantly reduced 2.29 times if they were managed with an insulin pump compared to those on multiple daily injections (95% CI, −4.41 to −0.17; *p*=0.03). This finding underscores the role of iatrogenic hypoglycaemia among children with T1DM [25, 27, 30].

### 4.2. Prediction Model

The primary aim of this study was to develop a prediction tool to identify children at risk of experiencing acute diabetic complications. Our final model comprised five predictors and had good discrimination at 6, 18, and 24 months (see Table 4 for ROC values). We then categorised our cohort into low-, moderate-, and high-risk groups using 95% sensitivity and 90% specificity cut-offs from the full study duration. Our analyses showed that participants from the high-risk group experienced *globally* worse outcomes. Compared to their low-risk peers, high-risk participants (1) were 11.7 times more likely to experience acute diabetic healthcare utilisation, (2) had a 35 times higher incidence rate ratio for repeated episodes of acute diabetic healthcare utilisation, and (3) experienced acute diabetic healthcare utilisation hundreds of days sooner. Using linear mixed models, we then showed that glycaemic control was persistently worse in the high-risk group over time and replicated this finding among each of the variables that comprise the model except for SES. Furthermore, 50% of these *high*-risk children required acute healthcare utilisation by 314 days; in comparison, this event took more than twice longer (765 days) among *moderate*-risk children. Our model did not perform well at 12 months (AUC = 0.67). The specific mechanism for this remains unclear. These analyses were adjusted for age, gender, and study duration.

Our results suggest that a simple suite of baseline characteristics could estimate future risk of acute healthcare utilisation. This could assist healthcare providers to effectively allocate resources towards children who are more likely to experience diabetic decompensation. Depending on the local clinical context and medical record setup, it might be possible to have a semi-automated system, and after further validation work, we plan to provide an open access interactive web app that would perform the necessary calculations. Secondly, we observed that similar children with T1DM can experience vastly different outcomes. In our cohort, diabetic outcomes varied by access to external support, and these supports had lasting effects on glycaemic control. This is reinforced by our finding that these factors have an ongoing relationship with HbA1c over time; early intervention may, therefore, change the trajectory of a child's diabetic outcomes into adulthood. Therefore, increasing access to (1) CGM, (2) insulin pump devices, and (3) support services while diligently following up on children who have missed appointments may reduce rates of acute diabetic decompensation, as well as improve long-term glycaemic control. Finally, our findings suggest that many factors affecting a child's diabetes remain out of their control, e.g.,socioeconomic status. In the context of rapid technological development such as hybrid closed loop insulin pumps [54, 55] and an increasing prevalence of T1DM [1, 56, 57], equity may play an increasingly important role in the future of outcomes in childhood T1DM.

## 5. Strengths and Limitations

There are some important strengths to our study. Firstly, we informed our analysis of predictive risk factors using a systematic literature search (Supplementary file 1), which identified 22 individual predictors; 11 of these were available and explored in our dataset. Secondly, our study contributes several novel findings; these include insights into the role of CGM, dynamic monitoring parameters, and various sociodemographic factors in the prediction of acute diabetic complications in children with T1DM. Finally, we contribute a simple, five-variable risk prediction tool that could be used to triage children with T1DM in resource-limited outpatient settings.

Despite this, our study also has several limitations. Firstly, our cohort was small (*n* = 103). Important predictive variables may have been omitted on this basis; we also did not adjust our analyses for multiple testing and may have committed type I error. Type II errors may have also been made; e.g., no cases of severe hypoglycaemia and DCJ involvement were recorded limiting our ability to explore this association. Secondly, our study is a pilot; we have not yet validated our tool temporally or with an external cohort. We postponed this study component due to SARS-COV-2 public health orders (lockdowns) in NSW and the CCLHD, which would likely confound the associations between our baseline risk factors and diabetic decompensation among children in our cohort. Our tool may, therefore, be unreliable in a pandemic setting. Research suggests that in populations of children with established T1DM, COVID-19 is associated with increased rates of hyperglycaemia but stable rates of hypoglycaemia [58]. Other environmental factors, including lifestyle, eating habits, and exercise, may also affect the incidence of acute complications. Our study is also retrospective, and comorbidities were noted from medical records instead of standardised assessments; although we have identified potential therapeutic targets, it is possible that interventions to optimise these risk factors may be ineffective in prospective, randomised studies. Further details not available in this study, such as reasons for missed appointment, may allow for greater accuracy in model building. We did not find an increased risk of complications in younger children, but this may have been related to our limited sample size and age distribution. Some of the variables in our final model also may not be reproducible in other settings. For example, a child's involvement with DCJ is specific to the Government of New South Wales, and we did not record the indication for their involvement. Although there are child welfare services in other states, this predictor would need to be validated before implementing the tool. Socioeconomic status was also measured by IRSAD quintiles, which the Australian Bureau of Statistics provided. To implement the model in another country, this variable would require modification. Finally, there are several alternative methods of model building which we did not pursue; future studies should look to combine individual patient data to attempt these approaches. Ultimately, this would enhance the accuracy and applicability of the model to diverse populations.

## 6. Conclusion

Acute healthcare utilisation occurs commonly among children and adolescents with T1DM due to diabetic decompensation. These preventable episodes carry significant morbidity and cost, but identifying children at high risk for these complications remains challenging. We provide a simple risk prediction model to estimate a child's risk for acute healthcare utilisation. Future feasibility testing and external and temporal validation of this model could establish it as a safe and cost-effective way to preventing diabetic decompensation in children and adolescents with T1DM.

## Figures and Tables

**Table 1:** Baseline characteristics by acute healthcare utilisation.

Characteristic	Children who experienced no AHU (*n* = 64)	Children who experienced AHU (*n* = 39)
Baseline age (years); median (IQR)	12 (24.0)	11 (23.0)
Gender (*N* (%))		
Male	27 (42.2)	22 (56.4)
Female	37 (57.8)	17 (43.6)
Duration of diabetes (years); median (IQR)	5 (10.5)	4 (9.0)
Age at T1DM diagnosis (years); median (IQR)	5.5 (12.0)	7 (15.0)
Baseline HbA1c (%); median (IQR)	8 (15.9)	8.6 (17.4)
Method of insulin administration (*N* (%))		
Multiple daily injections	26 (40.6)	24 (61.5)
Insulin pump	38 (59.4)	15 (38.5)
Continuous glucose monitoring (CGM) device (*N* (%))		
No	28 (43.8)	27 (69.2)
Yes	36 (56.3)	12 (30.8)
Acute healthcare utilisation in previous 12 months (*N* (%))		
None	58 (90.6)	24 (61.5)
1 event	4 (6.3)	5 (12.8)
≥2 events	2 (3.1)	10 (25.6)
Missed clinic visits in previous 6 months (*N* (%))		
None	59 (92.2)	25 (64.1)
1 visit	4 (6.3)	7 (18.0)
≥2 visits	1 (1.6)	7 (18.0)
Number of neuropsychiatric comorbidities (*N* (%))		
None	49 (76.6)	24 (61.5)
1 or 2	14 (21.9)	12 (30.8)
≥3	1 (1.6)	3 (7.7)
Involvement with DCJ (*N* (%))		
No involvement, ever	60 (93.8)	31 (79.5)
Previous involvement	2 (3.1)	0 (0.0)
Current involvement	2 (3.1)	8 (20.5)
SES, by IRSAD quintiles (*N* (%))		
1st quintile (most disadvantaged)	7 (10.9)	10 (25.6)
2nd quintile	25 (39.1)	10 (25.6)
3rd quintile	12 (18.8)	9 (23.1)
4th quintile	15 (23.4)	7 (18.0)
5th quintile (least disadvantaged)	5 (7.8)	3 (7.7)
Study duration (days); median (IQR)	563 (1,064)	540.5 (647.0)

AHU, acute healthcare utilisation; IQR, interquartile range; T1DM, type 1 diabetes mellitus; HbA1c, glycosylated haemoglobin; DCJ, Department of Communities and Justice; SES, socioeconomic status; IRSAD, Index of Relative Socioeconomic Advantage and Disadvantage.

**Table 2:** Univariate relationship between predictors, diabetic ketoacidosis, severe hypoglycaemia, and acute healthcare utilisation.

Individual predictors	Diabetic ketoacidosis (*n* = 20)	Severe hypoglycaemia (*n* = 9)	Acute healthcare utilisation (*n* = 39)
	*β* (95% CI)	*β* (95% CI)	*β* (95% CI)
Baseline age (years)	0.03 (−0.13 to 0.18)	0.11 (−0.11 to 0.34)	−0.01 (−0.13 to 0.11)
Female gender	−0.12 (−1.10 to 0.86)	−0.35 (−1.73 to 1.02)	−0.57 (−1.38 to 0.23)
Duration of diabetes (years)	−0.02 (−0.15 to 0.11)	0.03 (−0.14 to 0.21)	−0.07 (−0.18 to 0.04)
Age at T1DM diagnosis (years)	0.04 (−0.09 to 0.18)	0.05 (−0.13 to 0.23)	0.06 (−0.05 to 0.17)
Baseline HbA1c (%)	**0.57 (0.24 to 0.90)**	0.08 (−0.33 to 0.49)	**0.40 (0.11 to 0.69)**
Insulin pump	−0.07 (−1.05 to 0.90)	**−2.29 (−4.41 to −0.17)**	**−0.85 (−1.66 to −0.03)**
CGM device (yes)	**−1.17 (−2.27 to −0.07)**	−1.21 (−2.83 to 0.41)	**−1.06 (−1.90 to −0.22)**
Acute healthcare utilisation in the previous 12 months (ref: none)			
1 event	0.84 (−0.88 to 2.56)	0.29 (−1.93 to 2.51)	1.11 (−0.29 to 2.50)
≥2 events	**3.19 (1.71 to 4.67)**	−0.03 (−2.22 to 2.16)	**2.49 (0.90 to 4.08)**
Number of missed clinic visits in the previous 6 months	0.40 (−0.03 to 0.82)	0.40 (−0.10 to 0.89)	**1.01 (0.27 to 1.76)**
Number of neuropsychiatric comorbidities (ref: none)			
1	0.92 (−0.13 to 1.97)	−0.07 (−1.74 to 1.59)	0.56 (−0.35 to 1.47)
≥2	0.63 (−1.72 to 2.98)	1.31 (−1.10 to 3.73)	1.81 (−0.50 to 4.13)
Active DCJ case (ref: no involvement)	**2.64 (1.16 to 4.11)**	Nil cases	**2.05 (0.44 to 3.66)**
SES, IRSAD quintiles (ref: 1st quintile)			
2nd quintile	−0.97 (−2.30 to 0.36)	−1.26 (−3.16 to 0.63)	**−1.27 (−2.49 to −0.06)**
3rd quintile	−1.19 (−2.76 to 0.39)	−0.25 (−2.00 to 1.49)	−0.64 (−1.94 to 0.65)
4th quintile	−1.70 (−3.46 to 0.06)	−1.50 (−3.87 to 0.86)	−1.12 (−2.44 to 0.20)
5th quintile	0.10 (−1.65 to 1.84)	Nil cases	−0.87 (−2.59 to 0.86)
BMI *Z*-score (percentile)	−0.55 (−2.42 to 1.33)	−1.02 (−3.43 to 1.39)	−0.68 (−2.22 to 0.86)
Predictors for children with CGM device			
Baseline time in range (%)	−0.07 (−0.15 to 0.02)	−0.08 (−0.19 to 0.04)	−0.04 (−0.09 to 0.01)
Baseline wear time (%)	−0.01 (−0.07 to 0.05)	0.03 (−0.09 to 0.16)	0.00 (−0.05 to 0.04)
Baseline average number of calibrations per day	−0.06 (−0.34 to 0.22)	−0.47 (−1.34 to 0.41)	−0.11 (−0.31 to 0.09)
Predictor for children with no CGM device			
Baseline number of BGM checks per day	−0.13 (−0.41 to 0.16)	−0.07 (−0.43 to 0.29)	−0.12 (−0.36 to 0.13)
Predictors for children with insulin pump			
Baseline average number of days between set changes	0.27 (−0.26 to 0.80)	−0.44 (−2.55 to 1.67)	0.23 (−0.27 to 0.72)
Baseline average number of boluses per day	−0.40 (−0.83 to 0.02)	0.27 (−0.43 to 0.96)	**−0.40 (−0.77 to −0.03)**

CI, confidence interval; T1DM, type 1 diabetes mellitus; HbA1c, glycosylated haemoglobin; CGM, continuous glucose monitoring; DCJ, Department of Communities and Justice; SES, socioeconomic status; IRSAD, Index of Relative Socioeconomic Advantage and Disadvantage; BMI, body mass index. Bolded values indicate a *p* value <0.05.

**Table 3:** Multivariable relationship of parsimonious multivariable model.

	Acute healthcare utilisation	
	*β* (95% CI)	*p*
CGM device (yes vs. no)	−0.86 (−1.92 to 0.21)	0.12
Acute healthcare utilisation in the previous 12 months (ref: none)		
1 event	**1.80 (0.07 to 3.53)**	**0.04**
≥2 events	**2.87 (0.52 to 5.22)**	**0.02**
Number of missed clinic visits in the previous 6 months (ref: none)		
1	0.70 (−1.02 to 2.43)	0.43
≥2	**2.38 (0.06 to 4.70)**	**0.04**
Active involvement with DCJ (ref: no involvement)	1.61 (−0.26 to 3.49)	0.09
SES, IRSAD quintile (ref: 1st quintile)		
2nd quintile	−1.16 (−2.71 to 0.38)	0.14
3rd quintile	−0.65 (−2.23 to 0.93)	0.42
4th quintile	−0.47 (−2.06 to 1.13)	0.57
5th quintile	−2.26 (−5.03 to 0.52)	0.11

CI, confidence interval; CGM, continuous glucose monitoring; DCJ, Department of Communities and Justice; SES, socioeconomic status; IRSAD, Index of Relative Socioeconomic Advantage and Disadvantage. Multivariable analysis controlled for study duration. Bolded values indicate significance (*p* < 0.05).

**Table 4:** Discriminatory utility of parsimonious multivariable model.

	AHU at 6 months	AHU at 12 months	AHU at 18 months	AHU at 24 months	AHU for full study period
AUC (95% CI)	0.79 (0.64–0.94)	0.67 (0.55–0.78)	0.79 (0.69–0.89)	0.78 (0.68–0.89)	0.81 (0.72–0.90)
Sensitivity	0.69	0.61	0.72	0.70	0.68
Specificity	0.78	0.77	0.75	0.76	0.84

AHU, acute healthcare utilisation; AUC, area under the receiver operating characteristic curve.

**Table 5:** Cox hazard ratios of parsimonious multivariable model.

	Parsimonious multivariable model	
Model group	Hazard ratio (95% CI)	*p*
Low risk	—	—
Moderate risk	3.51 (0.80–15.38)	0.10
High risk	11.73 (2.75–49.99)	<0.01
Proportional hazard assumption		0.40

**Table 6:** Negative binomial regression of the number of acute healthcare utilisation episodes on risk group, adjusted for age, sex, baseline HbA1c, and study duration.

	*β* (95% CI)	IRR (95% CI)	*p*
Moderate risk	1.94 (0.31 to 3.56)	6.94 (1.37 to 35.26)	0.02
High risk	3.57 (1.87 to 5.27)	35.51 (6.48 to 194.55)	<0.01
Age (years)	0.1 (−0.04 to 0.24)	1.1 (0.96 to 1.27)	0.17
Female sex	−0.36 (−1.19 to 0.46)	0.7 (0.3 to 1.59)	0.39
Baseline HbA1c	0.01 (−0.3 to 0.32)	1.01 (0.74 to 1.38)	0.94
Chibar	91.64	—	<0.01

## Data Availability

The data that support the findings of this study are available on request from the corresponding author. The data are not publicly available due to privacy or ethical restrictions. Link recordings are not publicly available 12 months since the conference date.
