# Supplementary material for: Development of a Multivariable Risk Prediction Tool to Predict Adverse Outcomes among Children with Type 1 Diabetes: A Pilot Study
Source: Pediatr Diabetes. 2024 May 20;2024:8335604. doi: 10.1155/2024/8335604 (PMC12016770; doi:10.1155/2024/8335604)
Supplement: Supplementary Materials — are provided on the following electronic link. [file 8335604.f1.docx]

**Appendices**

**Appendix A1:** Comparison of CCLHD cohort with existing literature for select risk factors associated with acute diabetic complications

**Appendix A2:** Relationship between select risk factors and Diabetic Ketoacidosis in the literature

**Appendix A3:** Relationship between select risk factors and Diabetic hypoglycaemia in the literature

**Appendix A4:** Relationship between select risk factors and acute healthcare utilisation in the literature

**Appendix A5:** Definitions and data sources of outcomes and predictors

**Appendix A6**: Mixed models results for HbA1c by TREACLE risk group

**Appendices A7-A9:** Mean HbA1c trends by CGM wear (A7), DCJ involvement status (A8) and previous acute healthcare utilisation (A9).

**Appendix A1. Comparison of CCLHD cohort with existing literature for select risk factors associated with diabetic ketoacidosis, severe hypoglycaemia and acute diabetes-related healthcare utilisation in children and adolescents with type 1 diabetes mellitus.**

|  | **Diabetic ketoacidosis** | |
| --- | --- | --- |
|  | **CCLHD univariate effect size (95% CI)** | **Literature effect size range** |
| **Age** | OR: 1.03 (0.88 to 1.19) | OR: 1.02 - 2.21 |
| **Sex** | OR: 0.89 (0.33 to 2.35) | OR: 1.01 - 1.53 (Female) |
|  | OR: 1.12 (0.42 to 2.99) | OR: 0.68 - 1.41 (Male) |
| **Duration** | OR: 0.98 (0.86 to 1.11) | OR: 1.03 - 2.83 |
| **HbA1c** | OR: 1.77 (1.27 to 2.47) | OR: 1.44 (continuous %) |
|  | OR: 0.46 (0.12 to 1.72) | OR: 0.09 (<7.5 Vs >7.5) |
| **CSII** | OR: 0.93 (0.35 to 2.47) | OR: 0.79 |
| **AHU** | OR: 8.92 (2.97 to 26.84) | OR: 6.88 |
| **Psychiatric comorbidity** | OR: 2.42 (0.88 to 6.63) | OR: 1.80 |
| **SES** | OR: 1.10 (0.19 to 6.29) | OR: 1.66 |
|  |  |  |
|  | **Severe hypoglycaemia** | |
|  | **CCLHD univariate effect size (95% CI)** | **Literature effect size range** |
| **Age** | OR: 1.12 (0.90 to 1.40) | OR: 0.98 |
| **Sex** | OR: 0.70 (0.18 to 2.79) | OR: 1.01 (Female) |
|  | OR: 1.42 (0.36 to 5.62) | OR: 1.02 (Male) |
| **Duration** | OR: 1.03 (0.87 to 1.23) | OR: 1.06 - 1.08 |
| **HbA1c** | OR: 1.48 (0.35 to 6.28) | OR: 2.32 (<8.0 Vs >8.0) |
|  |  |  |
| **CSII** | OR: 0.10 (0.01 to 0.84) | OR: 0.69 |
| **AHU** | OR: 1.13 (0.22 to 5.87) | ***No literature*** |
| **Psychiatric comorbidity** | OR: 1.24 (0.29 to 5.32) | OR: 0.94 |
| **SES** | OR: 0.22 (0.02 to 2.36)* | ***No literature*** |
|  |  |  |
|  | **Acute diabetes-related healthcare utilisation** | |
|  | **CCLHD univariate effect size (95% CI)** | **Literature effect size range** |
| **Age** | OR: 0.89 (0.40 to 1.98) | OR: 1.94 (<12 vs 12-18) |
|  | OR: 0.99 (0.88 to 1.12) | OR: 0.93 (Age at dx continuous) |
| **Sex** | OR: 1.77 (0.79 to 3.96) | OR: 1.12 - 2.58 (Male) |
|  |  |  |
| **Duration** | HR: 0.92 (0.84 to 1.01) | HR: 0.98 - 1.00 |
| **HbA1c** | OR: 0.30 (0.10 to 0.88) | OR: 0.19 (<7.5 vs >7.5) |
|  | OR: 1.49 (1.12 to 1.99) | OR: 1.63 (continuous) |
| **CSII** | OR: 0.43 (0.19 to 0.97) | ***No literature*** |
| **AHU** | HR: 4.85 (2.45 to 9.61) | HR: 2.94 to 3.74 |
| **Psychiatric comorbidity** | OR: 2.04 (0.86 to 4.86) | ***No literature*** |
| **SES** | IRR: 0.89 (0.31 to 2.51) | IRR: 0.45 (highest vs lowest) |

CCLHD = Central Coast Local Health District; CI = Confidence Interval, OR = Odds Ratio, HbA1c = Glycosylated Haemoglobin, CSII = Continuous Subcutaneous Insulin Infusion, AHU = Acute Healthcare Utilisation, SES = Socioeconomic status, HR = Hazard Ratio, IRR = Incidence Rate Ratio.

*IRSAD 4 Vs IRSAD 1; data for IRSAD 5 not available in this analysis.

**Appendix A2: Relationship between select risk factors and Diabetic Ketoacidosis in the literature.**

| **Diabetic Ketoacidosis** | | |
| --- | --- | --- |
| **Author, Year** | **Exposure definition** | **Effect size (95% CI)** |
| **AGE** | | |
| Mejia-Otero, 2020 | Baseline age, years | OR*: 1.02, 95%CI (0.98, 1.06) |
| Nielsen, 2019 | Age at onset (6-12y vs <6y) | OR: 1.21, 95%CI (0.85, 1.73) |
|  | Age at onset (>12y vs <6y) | OR: 1.35, 95%CI (0.81, 2.26) |
| Schwartz, 2014 | Younger age (<12 y vs 12-18 y) | OR*: 1.05, 95%CI (0.43, 2.54) |
|  | Younger age at diagnosis (<12 y vs 12-18 y) | OR*: 2.21, 95%CI (0.62, 7.82) |
| Semenkovich, 2019 | Age, years | *b* = –0.256, SE = 0.132 |
| **SEX** | | |
| Ampt, 2019 | Female gender | OR: 1.01, 95%CI (0.97, 1.24) |
| Mejia-Otero, 2020 | Female gender | OR*: 1.53, 95%CI (1.18, 1.98) |
| Semenkovich, 2019 | Female gender | *b* = –0.317, SE = 0.421 |
| Nielsen, 2019 | Male gender | OR: 0.68, 95%CI (0.52, 0.89) |
| Sayed, 2017 | Male gender | OR*: 1.40, 95%CI (0.74, 2.67) |
| Schwartz, 2014 | Male gender | OR*: 1.41, 95%CI (0.58, 3.44) |
| **DURATION OF DIABETES** | | |
| Mejia-Otero, 2020 | Duration of diabetes, years | OR*: 1.03, 95%CI (0.99, 1.06) |
| Nielsen, 2019 | Duration of diabetes, years | OR: 1.07, 95%CI (1.02, 1.13) |
| Sayed, 2017 | Duration of diabetes > 5 years | OR*: 2.83, 95%CI (1.42, 5.67) |
| Semenkovich, 2019 | Duration of diabetes, years | *b* = 0.212, SE = 0.056 |
| **GLYCOSYLATED HAEMOGLOBIN (HbA1c)** | | |
| Alassaf, 2019 | HbA1c (<7.5% vs ≥7.5%) | OR*: 0.09, 95%CI (0.01, 1.51) |
| Mejia-Otero, 2020 | HbA1c, % | OR*: 1.44, 95%CI (1.36, 1.53) |
| Semenkovich, 2019 | Baseline HbA1c, % | *b* = 0.380, SE = 0.100 |
| **CONTINUOUS SUBCUTANEOUS INSULIN INFUSION (CSII)** | | |
| Nielsen, 2019 | Treatment regimen (pump vs basal/bolus) | OR: 0.79, 95%CI (0.58, 1.08) |
| Semenkovich, 2019 | Baseline pump | *b* = 0.526, SE = 0.415 |
| **ACUTE HEALTHCARE UTILISATION IN PRECEDING 12 MONTHS** | | |
| Mejia-Otero, 2020 | Admitted prior year? | OR*: 6.88, 95%CI (5.27, 8.99) |
| **CLINIC ATTENDANCE** | | |
| Mejia-Otero, 2020 | Number of clinic visits | OR*: 0.93, 95%CI (0.83, 1.04) |
| Sayed, 2017 | Lack of regular clinic visits for follow-up | OR*: 7.93, 95%CI (3.56, 17.75) |
| **PSYCHIATRIC COMORBIDITY** | | |
| Sildorf, 2018 | Presence of any psychiatric disorder | OR = 1.80, 95%CI (1.18, 2.76) |
| **SOCIOECONOMIC STATUS** | | |
| Ampt, 2019 | SES (most disadvantaged vs least disadvantaged) | OR: 1.66, 95%CI (1.16, 1.88) |
|  | SES (most disadvantaged vs least disadvantaged - repeat admissions) | OR: 3.00, 95%CI (2.14, 4.20) |

CCLHD = Central Coast Local Health District; CI = Confidence Interval, OR = Odds Ratio, HbA1c = Glycosylated Haemoglobin, CSII = Continuous Subcutaneous Insulin Infusion, AHU = Acute Healthcare Utilisation, SES = Socioeconomic status, HR = Hazard Ratio, IRR = Incidence Rate Ratio.

*Unadjusted Odds ratio


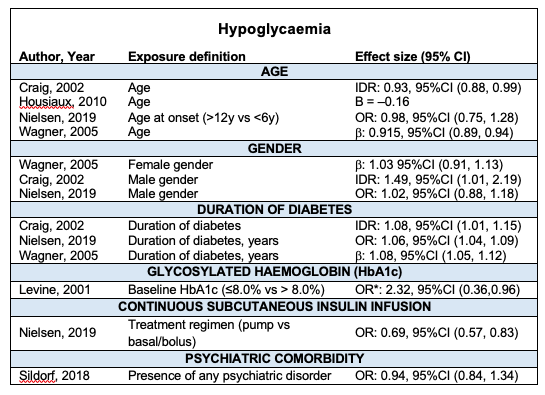

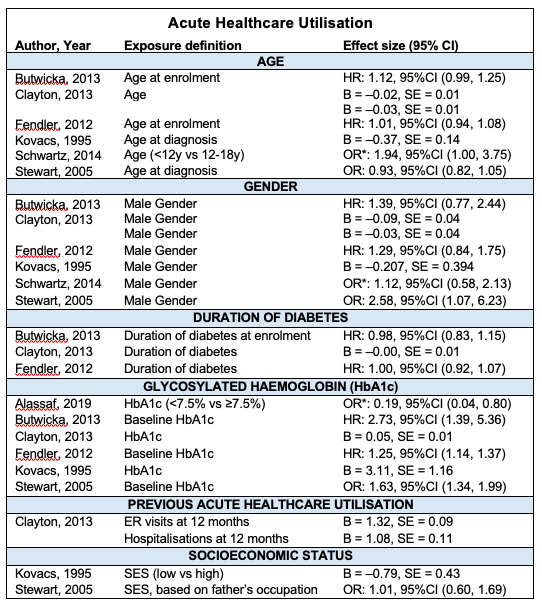


**Appendices A3 and A4: Relationship between select risk factors, Hypoglycaemia (A3) and Acute healthcare utilisation (A4) in the literature.**

CCLHD = Central Coast Local Health District; CI = Confidence Interval, OR = Odds Ratio, HbA1c = Glycosylated Haemoglobin, CSII = Continuous Subcutaneous Insulin Infusion, AHU = Acute Healthcare Utilisation, SES = Socioeconomic status, HR = Hazard Ratio, IRR = Incidence Rate Ratio

*Unadjusted odds ratio

**Appendix A5: Definitions and data sources of outcomes and predictors**

| **Variable** | **Type of data** | **Definition** | **EMR Data Source** | **Time of collection** |
| --- | --- | --- | --- | --- |
| Diabetic ketoacidosis (DKA) | Discrete | Number of DKA episodes recorded | Medical discharge notes, reason for admission | From time of first enrolled clinic visit to 31 December 2020 |
| Hyperglycaemia | Discrete | Number of hyperglycaemic episodes | Medical discharge notes, reason for admission | From time of first enrolled clinic visit to 31 December 2020 |
| Severe hypoglycaemia | Discrete | Number of severe hypoglycaemic episodes | Medical discharge notes, reason for admission | From time of first enrolled clinic visit to 31 December 2020 |
| Acute healthcare utilisation | Discrete | Sum of number of DKA, hyperglycaemic or severe hypoglycaemic episodes presented to emergency department (ED) or admitted to hospital | Medical discharge notes, reason for admission | From time of first enrolled clinic visit to 31 December 2020 |
| Baseline age, years | Continuous | Age in years at time of study enrolment | Demographics data | First enrolled clinic visit recorded since 1 January 2018 |
| Gender | Binary: 0 = Female; 1 = Male | Male or Female | Demographics data | First enrolled clinic visit recorded since 1 January 2018 |
| Age at T1DM diagnosis, years | Continuous | Age in years at time of T1DM onset | Diabetes educator clinic notes | First enrolled clinic visit recorded since 1 January 2018 |
| Duration of diabetes, years | Continuous | Duration in years from age of T1DM onset to first clinic visit at study enrolment | Diabetes educator clinic notes | First enrolled clinic visit recorded since 1 January 2018 |
| Baseline HbA1c, % | Continuous | Glycosylated haemoglobin value determined by DCA Vantage HbA1c analyser using a fingerstick blood sample | Diabetes educator clinic notes | At every clinic attended since first enrolled clinic visit |
| Method of insulin administration | Binary: 0 = Uses multiple daily injections; 1 = Uses insulin pump; | The status of how the patient receives insulin. They can receive insulin either by multiple daily injections (MDI) or an insulin pump, also known as a continuous subcutaneous insulin infusion (CSII). | Diabetes educator clinic notes | First enrolled clinic visit recorded since 1 January 2018 |
| Continuous monitoring glucose (CGM) device | Binary: 0 = Does not wear CGM; 1 = Wears CGM | The status of how a child performs glycaemic monitoring. ‘Does not wear CGM’ means the patient only monitors their glycaemic levels by fingerstick glucose tests or observed as infrequently wearing CGM. ‘Wears CGM’ means the patient wears CGM currently and consistently. | Diabetes educator clinic notes | First enrolled clinic visit recorded since 1 January 2018 |
| Acute healthcare utilisation in previous 12 months | Discrete | The number of any ED Presentation or hospital admission for DKA, hyperglycaemia or severe hypoglycaemia | Medical discharge notes | 12 months prior to their first enrolled clinic visit |
| Missed clinic visits in previous 6 months | Discrete | The number of clinic visits that were recorded as ‘did not attend’ | Diabetes educator clinic notes | 6 months prior to their first enrolled clinic visit |
| Number of neuropsychiatric comorbidities | Discrete | The number of neurodevelopmental or psychiatric disorders recorded in patient history | Diabetes paediatrician clinic notes | First enrolled clinic visit recorded since 1 January 2018 |
| Involvement with Department of Communities and Justice (DCJ) | Categorical:  0 = No DCJ involvement, ever;  1 = Previous history with DCJ involvement;  2 = Current/Active DCJ involvement | The patient’s history with Child Welfare services | Diabetes social worker clinic notes | First enrolled clinic visit recorded since 1 January 2018 |
| Socioeconomic status | Ordinal:  1 = 1^st^ Quintile, most disadvantaged;  2 = 2^nd^ Quintile;  3 = 3^rd^ Quintile;  4 = 4^th^ Quintile;  5 = 5^th^ Quintile, least disadvantaged | The patient’s home address postcode was entered into the Australian Bureau of Statistics 2016 Index of Relative Socioeconomic Advantage and Disadvantage (IRSAD) calculator to classify their IRSAD Quintile | Demographics data | First enrolled clinic visit recorded since 1 January 2018 |
| Study duration, days | Continuous | Number of days followed throughout the study | Diabetes educator clinic notes and medical discharge notes | From time of first enrolled clinic visit to 31 December 2020 |
| BMI Z-Score | Continuous | Body mass index measures adjusted for child age and sex | Diabetes educator clinic notes | First enrolled clinic visit recorded since 1 January 2018 |
| CGM specific variables | | | | |
| Baseline time in range, % | Continuous | Percentage of time the patient’s glycaemic values are not spent in hyperglycaemia or hypoglycaemia | CGM data was uploaded as an attachment to diabetes clinic notes | First enrolled clinic visit recorded since 1 January 2018 |
| Baseline wear time, % | Continuous | Percentage of time the patient wears their CGM device in a 14-day timeframe | CGM data was uploaded as an attachment to diabetes clinic notes | First enrolled clinic visit recorded since 1 January 2018 |
| Baseline average number of calibrations per day | Continuous | The average number of fingerstick calibrations carried out per day on the CGM device | CGM data was uploaded as an attachment to diabetes clinic notes | First enrolled clinic visit recorded since 1 January 2018 |
| No CGM specific variable | | | | |
| Baseline average number of blood glucose monitoring checks per day | Continuous | The average number of fingerstick blood glucose tests carried out per day, for children with no CGM device. This number is obtained from their blood glucose meter | Blood glucose meter data was uploaded as an attachment to diabetes clinic notes | First enrolled clinic visit recorded since 1 January 2018 |
| Insulin pump specific variables | | | | |
| Baseline average number of days between set changes | Continuous | The average number of days between insulin pump set changes | Insulin pump data was uploaded as an attachment to diabetes clinic notes | First enrolled clinic visit recorded since 1 January 2018 |
| Baseline average number of boluses per day | Continuous | The average number of insulin boluses delivered by insulin pump per day | Insulin pump data was uploaded as an attachment to diabetes clinic notes | First enrolled clinic visit recorded since 1 January 2018 |

**Appendix A6: Mixed models results for HbA1c by TREACLE risk group.** Analyses adjusted for sex, study duration and age.

|  |  | **Beta (95% CI)** |
| --- | --- | --- |
| **TREACLE Group** |  |  |
| *Moderate risk* |  | 0.62 (-0.13 to 1.37) |
| *High Risk* |  | 1.54 (0.72 to 2.36) |
|  |  |  |
| **Appointment** |  |  |
|  | *two* | 0.03 (-0.36 to 0.41) |
|  | *three* | 0.11 (-0.34 to 0.57) |
|  | *four* | 0.11 (-0.41 to 0.63) |
|  | *five* | 0.21 (-0.43 to 0.84) |
|  | *six* | 0.39 (-0.47 to 1.25) |
|  | *seven* | 0.26 (-0.79 to 1.32) |
|  | *eight* | -0.31 (-1.86 to 1.24) |
|  | *nine* | 2.81 (0.61 to 5) |
|  |  |  |
| **Moderate Risk** |  |  |
| **Appointment** |  |  |
|  | *two* | -0.05 (-0.55 to 0.44) |
|  | *three* | 0.02 (-0.57 to 0.61) |
|  | *four* | -0.08 (-0.75 to 0.59) |
|  | *five* | -0.12 (-0.93 to 0.7) |
|  | *six* | -0.72 (-1.78 to 0.33) |
|  | *seven* | -0.94 (-2.22 to 0.35) |
|  | *eight* | -0.77 (-2.59 to 1.06) |
|  | *nine* | -3.68 (-6.23 to -1.12) |
| **High Risk** |  |  |
| **Appointment** |  |  |
|  | *two* | 0.4 (-0.14 to 0.95) |
|  | *three* | 0.52 (-0.13 to 1.18) |
|  | *four* | 0.05 (-0.69 to 0.79) |
|  | *five* | -0.09 (-0.96 to 0.79) |
|  | *six* | -0.68 (-1.79 to 0.42) |
|  | *seven* | -0.28 (-1.62 to 1.07) |
|  | *eight* | 1.02 (-0.91 to 2.95) |
|  | *nine* | *No results* |
|  |  |  |
| **Age** |  | 0.13 (0.05 to 0.21) |
| **Female Sex** |  | 0.42 (-0.11 to 0.94) |

**Appendix A7: Mean HbA1c trends by CGM wear**

**Appendix A9: Mean HbA1c trends by previous acute
healthcare utilisation**

**Appendix A8: Mean HbA1c trends by DCJ involvement status**

Appendices A7, A8 and A9 are mixed-effects models illustrating the trend of mean HbA1c over time with continuous glucose monitoring (CGM) wear (A7), child welfare involvement (DCJ) (A8) and previous acute healthcare utilisation (AHU) in the last 12 months (A9) as exposures, respectively. A7 shows children who wore no CGM at study enrolment had a persistently higher mean HbA1c (approximately 1%) for four appointments compared to children who wore CGM. A9 shows that children with multiple (≥2) AHU episodes in the 12 months preceding baseline had higher mean HbA1c, while A8 shows that children with active DCJ involvement have a higher HbA1c over time. However, both A8 and A9 are not statistically significant due to the small sample sizes created through group partition (data not presented).
